# Detection and serotyping of dengue viruses in febrile patients consulting at the New-Bell District Hospital in Douala, Cameroon

**DOI:** 10.1371/journal.pone.0204143

**Published:** 2018-10-03

**Authors:** Francine Berlange Sado Yousseu, Fredy Brice Simo Nemg, Sandra Ateutchia Ngouanet, Franck Martin Obam Mekanda, Maurice Demanou

**Affiliations:** 1 Department of Virology, National Reference Laboratory for Chikungunya and Dengue, Centre Pasteur of Cameroon (CPC), Yaoundé, Centre, Cameroon; 2 Department of Biomedical Sciences, University of Dschang, Dschang, West, Cameroon; CEA, FRANCE

## Abstract

Arboviruses are a major public health problem worldwide and are predominantly present in intertropical areas. Chikungunya, dengue and zika viruses have been implicated in recent epidemics in Asia, America and Africa. In Cameroon, data on these viruses are fragmentary. The purpose of this study was to determine the frequency of detection of these three viruses in febrile patients in Douala, Cameroon. A cross-sectional and descriptive study was conducted from March to April 2017 at the New-Bell District Hospital in Douala. Blood samples were collected from febrile patients and tested for malaria infections using Rapid Diagnostic test. Plasma harvested was later analyzed for the presence of chikungunya, dengue and zika viruses by a Trioplex real-time RT-PCR at Centre Pasteur of Cameroon. A total of 114 participants were included, of which 63.2% were females, reflecting a sex ratio (female/male) of 1.7. The median age was 26 years, range [0.25–81]. Eight (7%) of the 114 participants were infected with Dengue virus (DENV) among which 5 were identified as serotype 1. No cases of infection by either Zika virus or Chikungunya virus were detected. Three cases of dengue-malaria co-infection (13%) were recorded. No association was found between socio-demographic factors and dengue infection. The phylogenetic analysis of the partial envelope E gene showed that all the five DENV serotype 1 samples belonged to subtype V, similarly to strains from West African countries, particularly those from Nigeria, Senegal and Côte d’Ivoire. This study showed the circulation of DENV serotype 1 in febrile patients and raises the alarm for the establishment of a sustained surveillance system to detect cases and prevent potential outbreaks in Cameroon. The existence of dengue-malaria co-infections suggests that surveillance of arboviruses should not be limited to febrile, non-malarial cases.

## Introduction

Vector-borne diseases are the leading cause of morbidity and mortality in the world [1]. This is the case with arbovirus infections. These viruses are transmitted by infected arthropods. The majority of arboviruses that infect humans are classified into different families; *Nairoviridae (Crimean-Congo hemorrhagic fever virus*, *etc*.*)*, *Peribunyaviridae (Bunyamwera virus*, *Bwamba virus*, etc.), *Phenuiviridae* (Rift Valley fever virus, etc.), *Flaviviridae* (dengue virus—DENV, zika virus—ZIKV, etc.) and *Togaviridae* (chikungunya virus -CHIKV, equine encephalitis viruses, etc.). They are single-stranded, enveloped viruses with a ribonucleic acid (RNA) of positive-polarity [2].

The clinical diagnosis of arboviroses is difficult because of the absence of specific symptoms and due to its resemblance with other diseases characterized by the presence of fever such as malaria and typhoid fever [3]. Biological diagnosis can be made either in early stages of the disease (during the first few days) by the search for the virus or its constituents; or in later stages (7 days or more after the onset of clinical signs) by screening for antibodies against arboviruses with serological assays [4,5].

Dengue, zika and chikungunya viruses are mainly present in intertropical zones. These viruses have been implicated in recent epidemics in Asia, Africa and America with many complications such as chronic arthritis, hemorrhagic and encephalic syndromes in Asia and America [6,7]. In Africa, during the 2016/2017 dengue epidemics in Burkina Faso, cases of dengue hemorrhagic fever were reported [8] and 12 087 suspected cases including 7418 probable cases, and 20 deaths were recorded (case fatality rate 0.2%) [9]. The study by Herrera *et al*. (2017) in Nigeria and Senegal on febrile patients showed an anti-zika immunoglobulin M (IgM) seroprevalence of 6.2% and 4/387 cases of Zika virus detection using RT-PCR assay [10].

In Cameroon, several studies have been conducted on arboviruses in the last two decades. In 2004, Ndip et al. carried out a study in Tiko and Buea clinics among febrile patients tested negative for malaria and typhoid. Of 234 patients tested, 47% had anti-CHIKV antibodies and 47% had antibodies against some *Flaviviruses* such as dengue viruses 1–4, Yellow fever virus and West Nile virus [11]. Another study was performed by an American team (during an HIV survey) on the distribution of arboviruses of the families of *Flaviviridae*, *Togaviridae* and *Bunyaviridae* among adults in rural areas of southern Cameroon. They found that 12.5% were positive for DENV serotype 2 and 46.5% for CHIKV antibodies out of 256 participants [12]. A survey carried out in a rural area of the Western part of Cameroon in 2007 on 105 volunteers indicated that 51.4% had anti-CHIKV IgM and 39% anti-CHIKV IgG [13]. A study conducted by Demanou et al. in 2006/2007 based on the detection of anti-dengue IgM and IgG in three main towns of Cameroon, showed a seroprevalence of 61.4% IgG and 0.3% IgM in Douala; 24.2% IgG and 0.1% IgM in Garoua and 9.8% IgG and no IgM in Yaoundé [14]. Several other studies have been performed and showed varying proportions of arboviruses between 5% and 34.2% with serologic assays [15,16].

It appears from the literature above that previous studies have focused only on serologic tests with their known limitations because a negative test result would not rule out infection with the virus(es). On the other hand, very few studies have, however, enabled the detection of arboviruses using molecular techniques. Although real time PCR is not always realistic for viral detection as there is also viral isolation, we believe that before attempting viral isolation, it is important to first confirm acute infections. Therefore, this study aimed at confirming current circulation (or acute infections) of dengue, zika and chikungunya viruses in febrile patients consulting in a hospital setting in Douala using molecular assays.

## Materials and methods

### Description of the study site

**Fig 1:**
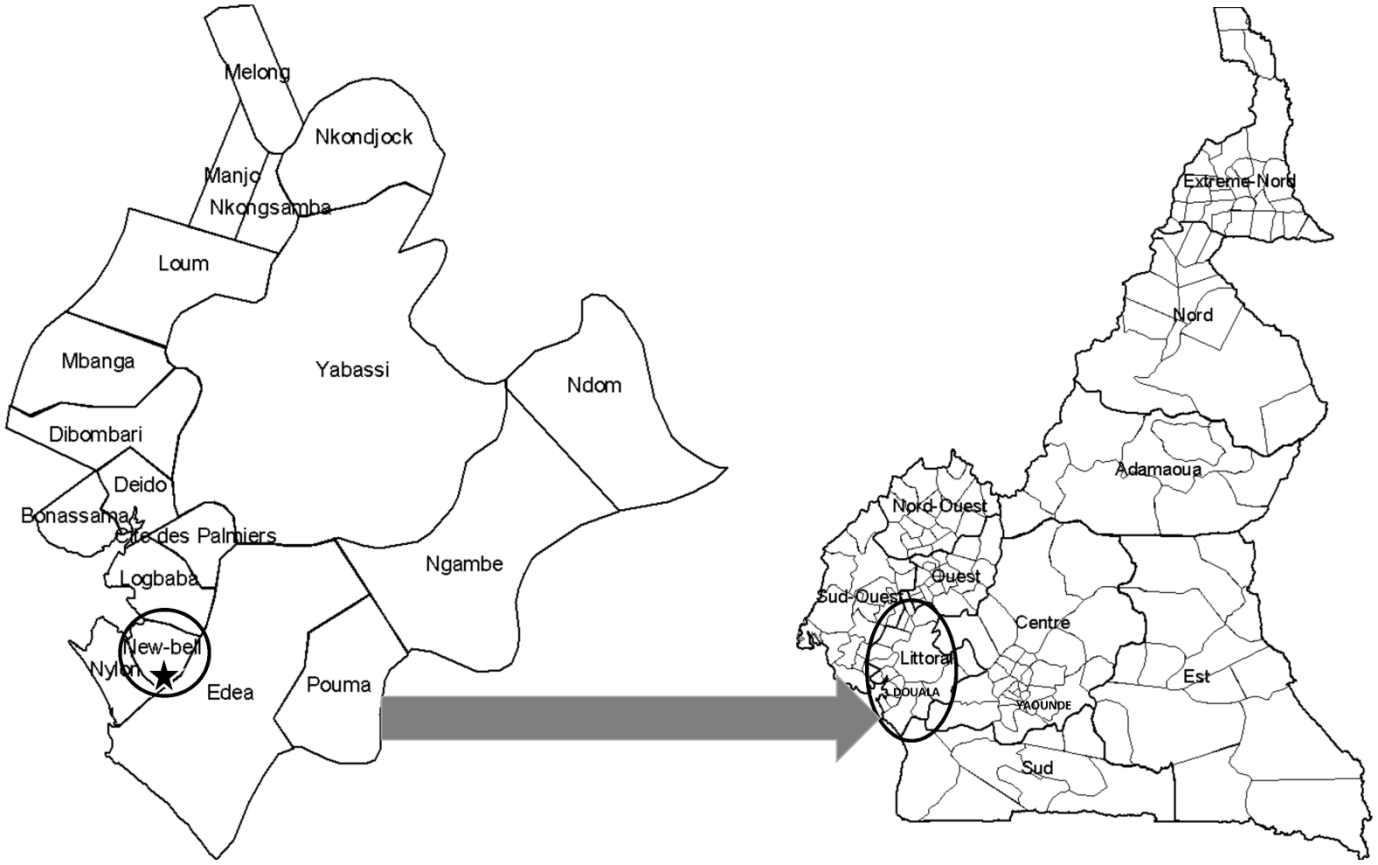
Location of New-Bell in Douala, Littoral Region of Cameroon. The study site is represented by the red star.

Douala, situated in the Littoral Region of Cameroon (Fig 1), is the economic capital and the largest city of the country with approximately 2.8 million inhabitants. This city is located between latitude 4° 2’ 53” N and longitude 9° 42’ 15” E and hosts the largest port of the country, which is also one of the largest in Central Africa, located on the shores of the Atlantic Ocean, at the bottom of the Gulf of Guinea, and at the mouth of the Wouri River. The climate of Douala is of the equatorial type, characterized by a constant temperature of about 26°C, and very abundant precipitation, particularly during the rainy season, from June to October. Air in this city is constantly saturated with 99% relative humidity in the rainy season and 80% in the dry season, which runs from November to May.

New-Bell is one of the urban districts of the Douala city. The management of garbage and flood waters has always been problematic in this district; which promotes the proliferation of mosquitoes, vectors of several infections including arboviruses [17].

### Recruitment of participants and collection of samples

A cross-sectional and descriptive study was conducted during two months (from March to April 2017) at the New-Bell District Hospital in Douala, Cameroon. During this period, all patients with acute fever (onset less than 7 days), associated with at least one of the following symptoms: muscle aches, headache, arthralgia, and rash were submitted to a standardized questionnaire (S1 File). Bleeding was not included in symptoms because fever and bleeding are scarcely reported by clinicians and no case of hemorrhagic fever has been documented so far. Blood samples were then collected from consented participants into 5mL EDTA (ethylene diamine tetra-acetic acid) tubes. All the 114 collected samples were screened onsite for malaria infections with a rapid diagnostic test (RDT) and the remaining blood was immediately processed in order to obtain plasma, essential for subsequent analysis. All plasma specimens were stored at -20°C for 1–2 days at the Centre Pasteur of Cameroon (CPC) facilities in Douala, and transported on ice packs using the CPC shuttle to the Arbovirus Laboratory in Yaoundé the capital city, situated at about 300 km east, where they were stored at -80°C until testing.

This study was approved by the National Ethics Committee of Research for Human Health (*Comité National d’Ethique de la Recherche pour la Santé Humaine*, *CNERSH*) and thus was assigned the ethical clearance number 2017/03/881/L/CNERSH/SP (S2 File). All participants read and signed the informed consent form before enrollment to the study.

### Analysis of samples

#### Detection of dengue, zika and chikungunya viruses by real time PCR

Samples were tested during the period of May to July 2017 in the Arbovirus Laboratory of Centre Pasteur of Cameroon. CDC (Centers for Disease Control and prevention) Trioplex real-time RT-PCR assay was used for the simultaneous detection of DENV, ZIKV and CHIKV [18]. This assay’s principle is based on the use of double-marking hydrolysis probes (TaqMan^®^) with specific primers and fluorochromes. Prior to amplification with the molecular test, RNA was extracted from 140 μL of collected sample using the QIAamp Viral RNA Mini Kit (Qiagen laboratories). RNA extracts were eluted into 60 μL RNase/DNase free water. Following extraction, amplification was done using the SuperScript^®^ III Platinum One-step qRT-PCR enzyme kit (Invitrogen Life Science Technologies) and according to the protocol described by the Centers for Disease Control and prevention (CDC: Trioplex Real Time RT-PCR, 2017). A reaction volume of 25 μL contained 10 μL RNA extract, Rnase/Dnase free water, 2X Reaction mix, primers and probe mixture (DENV, CHIKV and ZIKV). Cycling conditions for the Trioplex reaction were as follow: reverse transcription at 50°c, 30 min; denaturation at 95°c, 2 min; 45 cycles at 95°c, 15 sec and 60°c, 1 min. Each run included no-template control (NTC), positive control for ZIKV, CHIKV and DENV (inactivated RNA for each virus) and Human specimen control (HSC) were used as control for RNA extraction. After amplification, the test was validated only if positive control for DENV, ZIKV and CHIKV are respectively positive only for DENV, ZIKV and CHIKV with a cycle threshold (CT) less than 38; the NTC should be negative for DENV, ZIKV and CHIKV and the HSC should only be positive for RNase P mix.

#### Dengue virus serotyping RT-PCR

Samples positive for dengue virus were then serotyped using a semi-nested RT-PCR as described previously by Chien et al. (2006). Briefly, the protocol involved two sequential amplifications where complimentary DNA (cDNA) is reverse transcribed from viral RNA present in the sample and amplified by PCR using C-prM primer set. The initial RT-PCR amplification was performed in a 50-μl reaction mixture containing 5 μl of RNA, and mD1 and D2 primers. The semi-nested PCR was performed using 5 μl of RT-PCR product from the initial reaction together with primer mD1 in combination with rTS1, mTS2, TS3, and rTS4 to identify the specific serotypes of dengue viral RNA in four separate reactions. After amplification, a 5-μl portion of each product was allowed to migrate on 2.5% agarose gel and identification of serotypes was based on the size of the fragments obtained; DENV-1 (208 bp), DENV-2 (119 bp), DENV-3 (288 bp), DENV-4 (260 bp) [19].

#### Dengue virus genotyping RT-PCR

A fragment of the partial envelope E gene of the dengue virus positive samples was amplified by RT-PCR as previously described [20] with some modifications. Briefly, 10μL of RNA was added to 40μL of PCR mixture containing 12 μL of water, 25μL of 2X PCR buffer mix, 0,5μL of E gene sense primer (d1s22: 5′-AGTTGTTAGTCTACGTGGAC-3′), and reverse primer (d1a17: 5′-CCAATGGCYGCTGAYAGTCT-3′) and 1μL of superscript III reverse transcriptase with platinum Taq High fidelity (Invitrogen-Thermo Scientific, USA). To improve the sensitivity, a semi-nested reaction was performed with primer d1a17 and d1s6 (5′-GGYTCTATAGGAGGRGTGTTCAC-3′). Briefly, 5μL of the first PCR product was added to 45μL of PCR mixture containing 40μL of water, 1,5μL of Mgcl2 at 50mM, 1μL of dNTPs at 10mM, 5μL of PCR buffer 10X, 0,5μL of forward (d1s6) and reverse (d1a17) primers, and 1μL of Taq DNA Polymerase (Invitrogen, USA). The expected 250 bp amplified partial E gene product was observed after electrophoresis in a 1.5% agarose gel.

#### Sequencing and phylogenetic analysis of dengue virus

PCR products were purified and sequenced using Big Dye Terminator cycle sequencing (Applied Biosystems, Foster City, USA) and semi-nested PCR primers according to the manufacturer instructions. Electrophoresis and data collection were done on an Applied Biosystems AB3100 genetic analyzer. Sequences were first aligned and edited using Sequence Navigator1 software (Applied Biosystems, Foster City, CA, USA). The consensus sequence was generated by removing low quality base peaks at the end of the chromatogram and correcting base pair mismatches.

Genotypes were determined by phylogenetic analysis of the envelope E gene by comparing the consensus sequence of each sample to the reference sequences of dengue virus serotype 1 as previously identified.

Phylogenetic analyses were performed using MEGA software version 6 which consisted in alignment of sequences using Clustal W algorithm [21]. Genetic distances were then inferred using the Kimura-2 parameter model and finally, a phylogenetic tree was generated by the neighbor-joining algorithm. The robustness of the phylogenetic tree was evaluated with 1000 bootstrap replicates. All consensus nucleotide sequences obtained in this study were submitted to GenBank Database and assigned the accession numbers MH802053 –MH802057.

### Statistical analysis

Statistical analysis was performed using SPSS version 22.0. Qualitative variables were expressed as proportions while median and interquartile ranges were used for quantitative variables. The Chi-2 test was used to search for associations between the presence of the dengue virus and the socio-demographic characteristics of the population studied. A difference was considered to be statistically significant at a p-value of less than 0.05. The degree of association between disease status and symptoms was assessed by calculating the Odds ratio framed by their 95% confidence intervals.

## Results

### Description of the socio-demographic characteristics of the participants

A total of 114 consenting participants were included. Of these, 42 (36.8%) were males and 72 (63.2%) were females reflecting a sex ratio of 1.7 in favor of females. The median age of the population was 26 years ranging from 3 months to -81 years of age. Table 1 shows the distribution of participants according to the following socio-demographic characteristics: gender, age group, educational level, marital status and occupation. The majority of the study participants were single (55.3%), 34.2% were in secondary school, 39.5% were unemployed and 40.4% were under 19 years of age (Table 1).

**Table 1:** Description of the socio-demographic characteristics of the participants.

Variables		Numbers	Percentages (%)
Sex	Male	42	63.2
	Female	72	36.8
	Total	114	100
Age range	[0–19]	46	40.4
	[20–39]	38	33.3
	[40–59]	16	14.1
	[60–79]	11	9.6
	≥80	3	2.6
	Total	114	100
Instruction	Unschooled	33	28.9
	Primary	32	28.1
	Secondary	39	34.2
	University	10	8.8
	Total	114	100
Marital status	Single	63	55.3
	Fiance	6	5.3
	Married	33	28.9
	Divorced	1	0.9
	Widow	11	9.6
	Total	114	100
Profession	Trader	12	10.5
	State worker	3	2.6
	Business worker	4	3.5
	Household	28	24.6
	unemployed	45	39.5
	Student (secondary)	5	4.4
	Student (university)	4	3.5
	Others	13	11.4
	Total	114	100

Concerning the level of knowledge of the study population with regard to these three arboviruses, only 3/114 (2.6%) participants had already heard of DENV, among which one had also heard about the ZIKV and CHIKV.

### Frequency of dengue, chikungunya and zika virus infections

Among the 114 samples tested for the presence of dengue, zika and chikungunya viruses by Trioplex real-time RT-PCR assay, only DENV was detected in 8 patients (7%). No cases of infection by either zika virus or chikungunya virus were detected.

**Fig 2:**
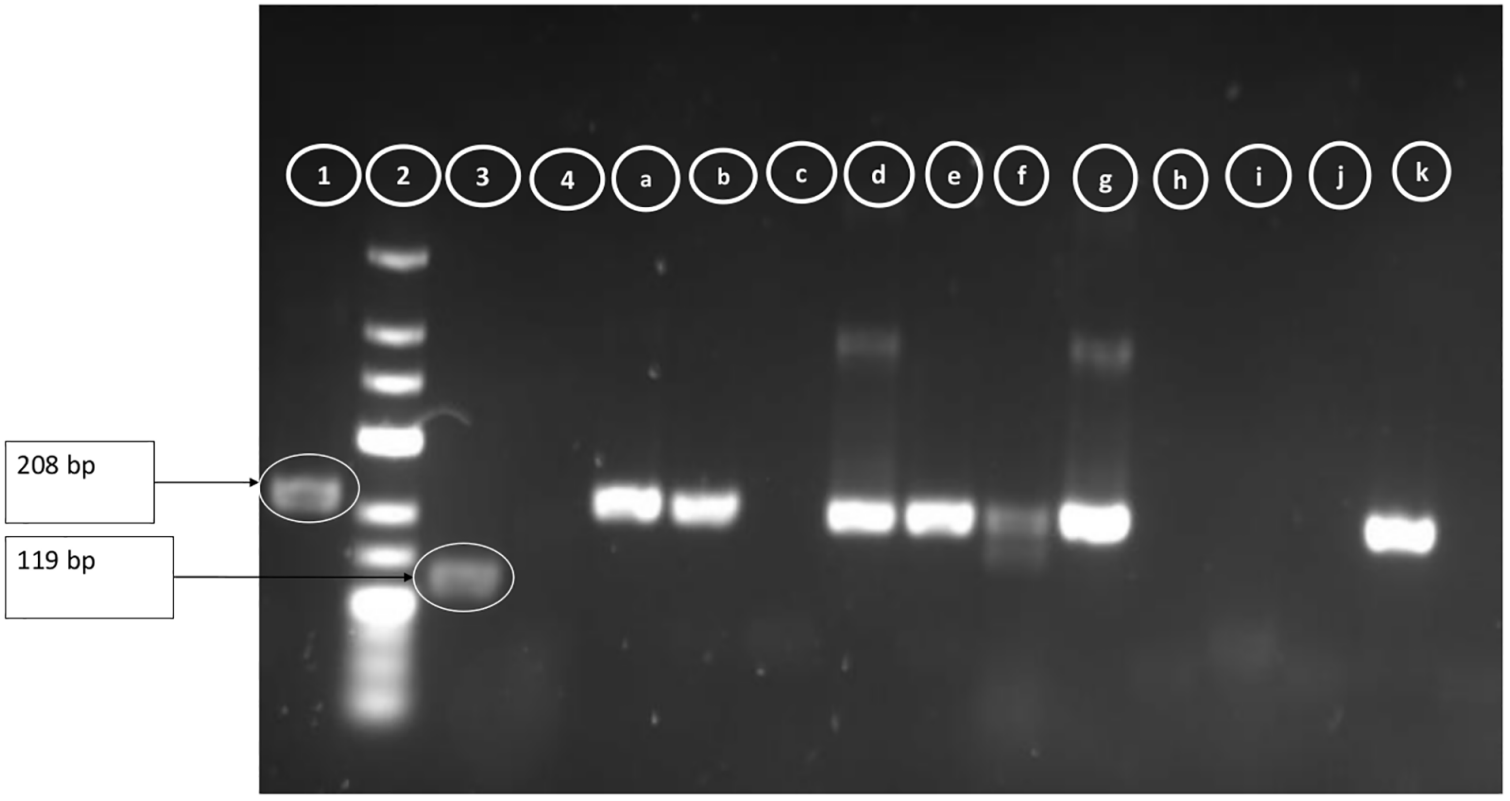
Image of PCR products obtained after migration on 1.5 agarose gel. 1 = Dengue 1 positive control; 2 = Molecular weight marker (100 bp); 3 = Dengue 2 positive control; 4 = Negative control; a,b,c,d,e,f,g,h,i,j,k = Samples.

Serotyping was possible in 5 of the 8 cases and all five were of dengue virus serotype 1 (Fig 2). The three positive samples with high Ct values (36–37) were not amplified by semi-nested PCR.

Table 2 summarizes the association between dengue virus infection and, demographic characteristics, population behavior and disease symptoms. The proportion of infected men (50%) was similar to that of infected women and the most represented age group among the infected participants was [20–39] years. No statistically significant association was noted between dengue virus infection and demographic characteristics (p-value> 0.05).

**Table 2:** Association between dengue virus infection and, socio-demographic characteristics, population behavior and disease symptoms.

		DENV positive patients (%)	DENV negative patients (%)	OR (CI 95%)	P value
Sex	Male	4 (50.0%)	38 (35.9%)	0.56 (0.13–3.36)	0.42
	Female	4 (50.0%)	68 (64.2%)		
Age range	[0–19]	2 (25.0%)	44 (41.5%)	1.30 (0.25–6.78)	0.56
	[20–39]	4 (50.0%)	34 (32.1%)		
	[40–59]	2 (25.0%)	14 (13.2%)		
	[60–79]	0 (0.0%)	11 (10.4%)		
	≥80	0 (0.0%)	3 (2.8%)		
Insecticides	Yes	4 (50.0%)	52 (40.1%)	1.04 (0.25–4.37)	0.96
	No	4 (50.0%)	54 (50.9%)		
Lodgings	Yes	5 (62.5%)	84 (79.3%)	0.44 (0.1–1.97)	0.27
	No	3 (37.5%)	22 (20.8%)		
Clothing	Exposed	3 (37.5%)	57 (53.8%)	1.94 (0.44–8.53)	0.37
	Not exposed	5 (62.5%)	49 (46.2%)		
Traveling	Yes	2 (25.0%)	15 (14.2%)	2.02 (0.37–10.97)	0.41
	No	6 (75.0%)	91 (85.9%)		
Temperature	<38°	0 (0.0%)	8 (7.6%)	1.08 (1.02–1.14)	0.42
	≥38°	8 (100.0%)	98 (92.5%)		
Duration of fever	1-5days	8 (100.0%)	104 (98.1%)	0.93 (0.88–0.98)	0.69
	6-7days	0 (0.0%)	2 (1.9%)		
Joint pain	Yes	4 (50.0%)	52 (49.1%)	1.04 (0.25–4.37)	0.96
	No	4 (50.0%)	54 (50.9%)		
Muscle aches	Yes	4 (50.0%)	39 (36.8%)	1.72 (0.41–7.26)	0.46
	No	4 (50.0%)	67 (63.2%)		
Vomiting	Yes	2 (25.0%)	31 (29.3%)	0.81 (0.15–4.22)	0.79
	No	6 (75.0%)	75 (70.8%)		
Headache	Yes	3 (37.5%)	46 (43.4%)	0.78 (0.18–3.45)	0.75
	No	5 (62.5)	60 (56.6%)		
Rash	Yes	1 (12.5)	6 (5.7%)	2.38 (0.25–22.62)	0.44
	No	7 (87.5%)	100 (94.3%)		

DENV: Dengue virus; OR: odds ratio; CI: confidence intervals.

With respect to some socio-demographic factors, clothing, non-use of insecticide and history of travel were associated with the presence of dengue virus (OR> 1). Similarly, some symptoms such as fever, joint pain, muscle pain and rash were associated with dengue virus infection but none of these associations are statistically significant (P> 0.05). All positive participants had an axillary temperature equal or more than 38°C, and the fever duration within 1–5 days. The commonest signs were fever, joint pain, muscle ache and headache. Only one positive participant presented rash (Table 2).

### Frequency of malaria and dengue/malaria co-infections

Of the 114 participants, 23 (20.2%) were positive for malaria RDT. Among the 23, three (13%) were also positive for DENV serotype 1.

### Genotyping of dengue virus

**Fig 3:**
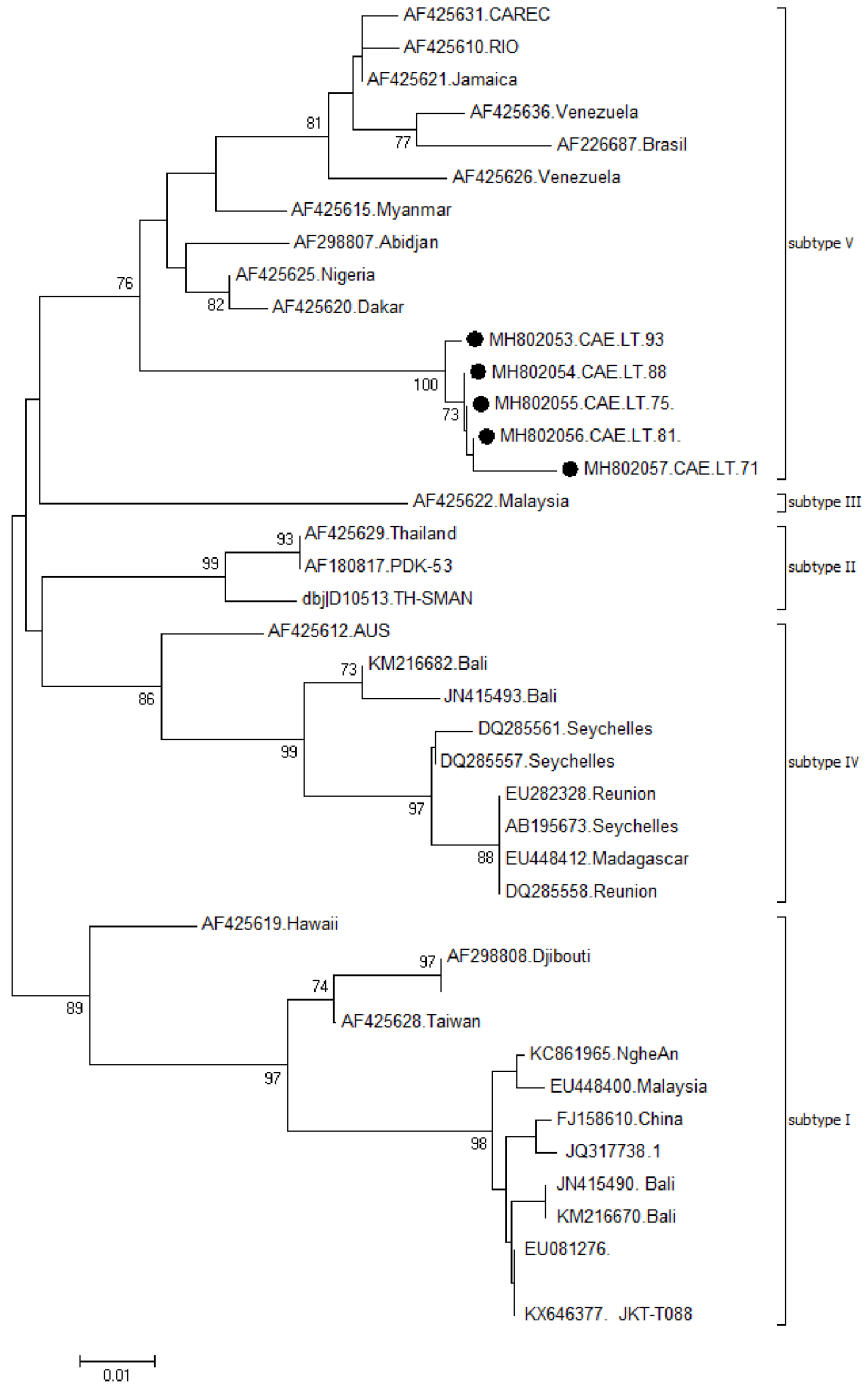
Phylogenetic tree of dengue virus serotype 1 strains from Cameroon based on partial envelope gene (211 bp) and their most related sequences from GenBank repository. Sequence from Cameroon are represented by solid circles (●). The tree was generated by neighbor joining alignment.

Among the 8 DENV positives samples, the partial envelope gene was generated for 5 (62,5%) samples. Fig 3 shows the estimated phylogeny of these sample with respect to relevant sequences available in Genbank. We observed that all the five samples grouped into subtype V of dengue 1 based on Goncalvez classification [22]. Cameroonian strains were closely related to West African strains particularly those from Nigeria, Senegal and Côte d’Ivoire.

## Discussion

This study is one of the first to ascertain the presence of dengue, zika and chikungunya genomes and not antibodies against these viruses, with detection of dengue virus serotype 1 (by Trioplex real-time RT-PCR and then a more conventional approach based on RT-PCR gel electrophoresis for serotyping) in Cameroon. During a two years study (2012/2013) carried out in both Central African Republic and Cameroon Demanou and colleagues failed to detect acute dengue cases, the main limitations of this study being the exclusion of positive malaria cases and the fact that the recruitment of patients rely on local and usually non motivated nurse and clinicians (unpublished data).

It is the second study that reports cases of dengue/malaria co-infection after the one conducted from 2012 to 2013 in the city of Yaoundé, Cameroon after testing retrospectively malaria positive cases [23]. The objective of this study was to determine the frequency of detection of DENV, ZIKV and CHIKV acute infections in febrile patients consulting at the New-Bell District Hospital in Douala.

The Centre Pasteur of Cameroon which was designated National Reference Laboratory for dengue, chikungunya and other arboviruses in 2015, has implemented various diagnostic techniques. Despite this, there is no nation-wide arbovirus surveillance system. The arbovirus team of CPC therefore initiated this sentinel surveillance in the city of Douala, Littoral region of Cameroon. It is noteworthy that several studies have been carried out on arboviruses in Cameroon in the last two decades. The first one focused on the identification of potential vectors of these viruses [24], and the others on the search for IgG and IgM antibodies [11,13,25]. The only few cases of detection of dengue viruses by RT-PCR (from Cameroon) were made abroad from travelers returning to their countries after holidays in Cameroon [26].

The eight cases (7%) of dengue detected by molecular methods, with 5 cases of serotype 1 confirm the silent circulation of the DENV-1 virus in Cameroon as described by Fokam *et al*. (2010) who obtained 2.50% DENV-1 in febrile patients by inhibition of haemagglutination and complement fixation [15]. The fact that only 7% of suspected dengue cases were confirmed is not a surprise. Demanou et al. reported an anti-DENV IgM prevalence of 0.3% in 2006 in Douala during a serosurvey involving more than 700 participants [14]. Also, a great percentage of negative results could be attributed to many other endemic diseases in Cameroon that present similar symptoms including malaria (20% in our study), leptospirosis, rickettsiosis and others not tested.

Despite this apparently low incidence of dengue, this result shows the importance of the implementation of a sustained arbovirus surveillance program in all regions of Cameroon. Circulation of DENV-1 is common in Central Africa although molecular characterization of the Cameroonian strains was not done. During a dengue outbreak in Angola in 2013, this serotype was suspected to be the exclusive cause of classical and uncomplicated dengue cases [27], even though few cases of other DENV serotypes were later detected retrospectively by Abreu et al. [28]. In addition to this study, the circulation of DENV serotype 4 was further suggested the following year, via the description of a DENV/CHIKV coinfection in a patient returning from Luanda [29].

The clinical signs observed were those of dengue like syndrome. No cases of dengue hemorrhagic fever (DHF) were reported. This observation corroborates that of Demanou et al. (2014) who highlighted the fact that despite the endemic circulation of DENV serotypes 1 and 2 (7 to 11% of the study population were infected by both serotypes), no DHF case has been reported in the country so far [14].

The malaria incidence of 20.2% (23/114) in Douala during March-April was relatively low when compared to 56.8% (179/315) recorded from children in Yaoundé (from February to April 2014), the capital city of Cameroon [30]. This low incidence could be explained by the sustained control efforts such as the use of insecticide treated bed nets and/or by the fact that malaria is more prevalent in children under five years of age, while in our study, all age groups were represented.

The frequency of dengue cases/malaria co-infections in our study was 13% (3/23) which is close to the 12.50% (3/24) observed in 2012/2013 in febrile patients in two hospitals of the capital city of Yaoundé, Cameroon [23]. Ayorinde *et al*. (2016), working on malaria and some arboviral infections in febrile patients visiting a health center in Simawa, Nigeria, reported 3% (2/60) dengue-malaria co-infections [31]. The socio-demographic characteristics of the population could explain this difference; Simawa is a rural community whereas Douala and Yaoundé are urban areas with high population densities and rapid development of activities, which are favorable to the development of vectors and eventually lead to arboviral transmission.

No factors were found associated with DENV infection because the association was not statistically significant (P> 0.05). This could be explained by the small number of positive samples. For this reason a longer-term monitoring program might be necessary.

The absence of circulation of CHIKV and ZIKV during the study period does not mean that these viruses are not present in the city of Douala. Serological evidence of ZIKV circulation has been shown by Gake *et al*. (2017), during a survey in blood donors, where they reported a 10% incidence of anti-zika IgG in the city of Douala [16]. And a strain of CHIKV was isolated in a febrile patient in May 2013 in Douala [32]. This negative result recorded during the present study could be due to the short duration of the study and/or the fact that we used standard volume RNA extraction of 140 μl. Also, concerning ZIKV, the fact that we tested only plasma instead of urine where the virus is more present or concentrated could explain the results as well. Indeed, studies have shown that large volume (1 mL) RNA extraction methods provide the highest sensitivity format; thereby increasing assay sensitivity by 0.5 logs and 0.7 logs genome copy equivalents per milliliter (GCE/mL) in serum and urine, respectively [33].

This study provides the first information on genome characterization of dengue virus serotype 1 from Cameroon. All strains from this study were found to cluster with subtype V similarly to West African strains. DENV-1 subtype V has well been found to circulate in Asia and America [20,22]. The genetic distance between Cameroon strains and the other African strains of subtype V was lower (92.8–95.0%) compared to strains from Asia and America of similar subtype (90.7–91.8%). This study shows the need to also suspect dengue fever and other arboviruses in case of fever. Doctors should therefore not be limited solely to the suspicion of malaria and typhoid fever while consulting a febrile patient. Sensitization of populations on the presence of these viruses should be a priority. Since general vaccination of the population against these arboviruses is not yet available, preventive measures should be encouraged.

This study provides data on dengue circulation and highlights the importance of a sustained surveillance system of these arboviruses in order to detect cases and prevent potential outbreaks in Cameroon and in Central Africa as a whole. The existence of dengue-malaria co-infections suggests that surveillance of arboviruses should not be limited to febrile, non-malarial cases as their clinical picture could be more severe than single infections [34].

## Supporting information

S1 File Standardize questionnaire used during the survey. (PDF)

S2 File Ethical clearance of the National Ethics Committee of Research for Human Health. (PDF)
