# Supplementary material for: Detection and serotyping of dengue viruses in febrile patients consulting at the New-Bell District Hospital in Douala, Cameroon
Source: PLoS One. 2018 Oct 3;13(10):e0204143. doi: 10.1371/journal.pone.0204143 (PMC6169880; doi:10.1371/journal.pone.0204143)
Supplement: S1 File — (PDF) [file pone.0204143.s001.pdf]

QUESTIONNAIRE

Structure sanitaire : Hôpital de District de New-Bell (HDN)

I. Identification du participant

Code questionnaire Nº ____________

1-Niveau scolaire : Non scolarisé ☐ Primaire ☐ Secondaire ☐ Universitaire ☐

2-Sexe : féminin ☐ masculin ☐

3-Quelle est votre tranche d'âge (ans) : 0 à 10 ☐ 11 à 21 ☐ 22 à 32 ☐ 33 à 43 ☐

44 à 54 ☐ 55 à 65 ☐ 65 et plus ☐

3--Situation matrimoniale : fiancé(e) ☐ marié(e) ☐ divorcé(e) ☐ Célibataire ☐

Veuf (ve) ☐

4-Profession : Commerçant(e) ☐ fonctionnaires ☐ Particuliers ☐ autres ____________

5-Ville de résidence (village/quartier) ____________

II. Aspect environnemental et connaissance du sujet

6-Avez-vous déjà entendu parler de ces arbovirus : Dengue ☐ Chikungunya ☐ Zika ☐

7-Dormez-vous sur une moustiquaire imprégnée ? Oui ☐ non ☐

8-Utilisez-vous des insecticides ? Oui ☐ non ☐

9-Autour de votre maison y'a-t-il : des réservoirs d'eau stagnante ☐

Pneus abandonnés ☐ des soucoupes de fleur ☐

10-Après chaque pluie, videz –vous les réservoirs d'eau qui peuvent se trouver autour de votre maison ? Oui ☐ non ☐

11-Quel est votre tenue vestimentaire habituelle : Exposée ☐ recouvrant tout le corps ☐

Autres ____________

12-Avez-vous voyagé ces deux dernières semaines oui ☐ non ☐

Si oui précisé ______________________________

III. symptômes majeurs

13-Température : ______________

14-Depuis combien de temps avez-vous la fièvre : 1 à 5 jours ☐

6 à 15 jours ☐ 15 jours et plus ☐

15-Avez-vous des: douleurs articulaires ☐ vomissements ☐

Douleurs musculaires ☐ Céphalées ☐

16-Avez-vous des éruptions cutanées ? Oui ☐ non ☐

IV. Fiche réservée au laboratoire

Date de collecte de l'échantillon : ________/ ________/ ________ heure : ________

Code : ________

Identification des virus et résultats GE/TDR

17- RNA du virus Dengue : présent ☐ absent ☐

Si présent quel(s) sérotype(s) 1 ☐ 2 ☐ 3 ☐ 4 ☐

18- RNA du virus Zika : présent ☐ absent ☐

19-Chikungunya présent ☐ absent ☐

20-Résultats GE/TDR : positif ☐ négatif ☐
