# Supplementary material for: Detection and serotyping of dengue viruses in febrile patients consulting at the New-Bell District Hospital in Douala, Cameroon
Source: PLoS One. 2018 Oct 3;13(10):e0204143. doi: 10.1371/journal.pone.0204143 (PMC6169880; doi:10.1371/journal.pone.0204143)
Supplement: S2 File — (PDF) [file pone.0204143.s002.pdf]

COMITE NATIONAL D'ETHIQUE DE LA RECHERCHE POUR LA SANTE HUMAINE

Arrêté N° 0977/A/MINSANTE/SESP/SG/DROS/ du 18 avril 2012 portant création, organisation et fonctionnement des comités d'éthique de la recherche pour la santé humaine au sein des structures relevant du Ministère en charge de la santé publique

N° 2017/03/881/L/CNERSH/SP

Yaoundé, le 17 mars 2017

Cnethique_minsante@yahoo.fr

LETTRE D'INFORMATION

Le Comité National d'Ethique de la Recherche pour la Santé Humaine (CNERSH), en sa session extraordinaire du 17 mars 2017, a examiné le projet de recherche intitulé: **«Etude des arbovirus: cas particuliers des virus Dengue, Chikungunya et Zika chez les patients fébriles reçus en consultation à l'hôpital de district de New-Bell»** soumis par **Madame SADO YOUSSEU Francine Berlange,** Investigateur Principal, étudiante à la Faculté des Sciences, Université de Dschang.

Le Comité a formulé les observations et suggestions suivantes :

- A la virgule près, c'est le même protocole de recherche que celui de l'étudiant **MATUNDU Emmanuel NDAM** et de l'étudiante **MBOCHE NGUIFFO Lydie Gaëlle,** tous étant encadré par le **Docteur DEMANOU Maurice**
- Est-ce que c'est le projet du professeur?
- L'étudiant doit apprendre à écrire un protocole de recherche
- Les 03 étudiants sont convoqués à la prochaine réunion d'évaluation des protocoles de recherche

Le Comité a émis un **avis favorable** sous réserve de l'intégration des suggestions faites par les membres du comité avant la délivrance de la clairance éthique.

Ampliations

- MINSANTE

Le président

Pr Lazare KAPTUE
